# Climate change and health: preparing future doctors for a changing world

**DOI:** 10.1097/MS9.0000000000003760

**Published:** 2025-08-27

**Authors:** Akshay Lohana, Shehdev Meghwar, Siddhant Kumar Yadav

**Affiliations:** a Liaquat University of Medical & Health Sciences, Jamshoro, Sindh, Pakistan; b Patan Academy of Health Sciences, Lalitpur, Nepal

Climate change is no longer an environmental issue; rather, it is an issue of human health that has moved from a distant possibility to a pressing reality. As a major global health threat, it affects every medical specialty and presents significant challenges to health systems worldwide^[1]^. It risks the health, happiness, and lives of innumerable people^[2]^. This makes it essential for medical professionals to be prepared. The idea that humanity faces an existential threat from climate change is becoming increasingly difficult to ignore^[1]^. Our article aligned with Titan’s 2025 guidelines^[3]^.

This problem is complicated with both direct and indirect health effects. Direct threats include frequent and intense weather events, such as floods, droughts, and wildfires. Indirect threats include air pollution, famines, the spread of diseases carried by insects, rising antibiotic resistance, and civil unrest^[1]^. These problems make vulnerable populations the most difficult to manage. Many medical students recognize the health risks associated with climate change, believing that human health is at risk and that the health sector is not ready to address them^[4,5]^.

Despite this widespread recognition, teaching about the health impacts of climate change is not fully integrated into medical school curricula^[4]^. Current efforts, such as pilot projects, specific courses, or learning objective catalogs, often embed climate-related content into existing formats, such as case discussions^[4,5]^. However, studies have shown that teaching focused specifically on this issue is uncommon, especially in non-Western countries^[4]^. This gap in education is especially troubling because medical students want more information on the topic^[5]^.

The current prevention and adaptation strategies in the health sector need to be expanded to better prepare future doctors. Medical professionals have a fourfold responsibility: to identify, prevent, and treat diseases related to climate change; collect and interpret relevant medical data; help reduce greenhouse gas emissions within their institutions; and act as leaders in society to encourage broader change^[5]^. The ongoing evolution of training requirements in Graduate Medical Education (GME) also emphasizes the need for a curriculum that is flexible enough to adapt to these new challenges^[6]^. Doctors of tomorrow will need to be equipped with both a solid foundation of medical knowledge and skills to address these growing global impacts^[4]^.

Therefore, a more inclusive approach is required. Medical education should aim for a deeper curriculum that extends beyond rote memorization to a real grasp of key concepts. This can happen through new teaching strategies or by weaving climate health into subjects, such as problem-based learning^[5]^. The curriculum should also hone students’ emotional intelligence to help them understand the complex ties among equity, sustainability, and health using methods such as ethical case studies. Collaborating with medical educators from the Global South, where climate change impacts are most severe, is also crucial^[4]^.

I believe that it is the duty of educational institutions to equip future physicians with these challenges. We need to teach them the science behind these issues, while also enabling them to become strong advocates for their patients’ health and the health of the planet. The future of medicine and humanity is deeply connected to our capacity to confront existential threats posed by climate change. By acting now to reform medical education, we can ensure that future doctors are prepared to lead the way in address the global health crisis.

## Data Availability

Not applicable.

## Peer and provenance statement

Unsolicited and externally peer-reviewed.
